# Editorial: Molecular and Cellular Effectors in the Resolution of Inflammation

**DOI:** 10.3389/fimmu.2022.938819

**Published:** 2022-05-26

**Authors:** Amiram Ariel, Sylvain Perruche, Sylvaine You, János G. Filep

**Affiliations:** ^1^ The Department of Biology, The Faculty of Natural Sciences, University of Haifa, Haifa, Israel; ^2^ The Departments of Human Biology, The Faculty of Natural Sciences, University of Haifa, Haifa, Israel; ^3^ Univ. Bourgogne Franche-Comté, INSERM, EFS BFC, UMR1098 RIGHT, Interactions Hôte-Greffon-Tumeur/Ingénierie Cellulaire et Génique, Besançon, France; ^4^ MED‘INN’Pharma, Besançon, France; ^5^ Université Paris Cité, CNRS, INSERM, Institut Cochin, Paris, France; ^6^ Department of Pathology and Cell Biology, University of Montreal, Montreal, QC, Canada; ^7^ Research Center, Maisonneuve-Rosemont Hospital, Montreal, QC, Canada

**Keywords:** resolution of inflammation, myeloid cells, lymphocytes, soluble mediators, intracellular signaling

Acute inflammation is broadly defined as a protective response of the organism to invading pathogens and tissue injury. The reaction should ideally be localized and self-limited, enabling the elimination of pathogens and damaged cells, and resolve on its own, leading to tissue repair and restoration of normal tissue and organ function (1). Uncontrolled or excessive inflammation or failure of the initial response to resolve in a timely manner leads to nonresolving inflammation, which is often chronic, or transient and relapsing (2). This ongoing low-grade inflammation is thought to fail to activate the integrated process of resolution (3), which is increasingly being recognized as a critical component of numerous prevalent human diseases, including atherosclerosis, arthritis, pulmonary diseases, autoimmunity, diabetes and cancer (2, 4). The inflammatory response is a complex process, heterogeneous in nature and depends on the type of disease and organ in which it occurs. The sequence of events and signaling circuits activated during the initiation and resolution phase have received considerable attention, and are summarized in guiding maps for inflammation (5) and resolution pathways (6). Resolution is an active process governed by endogenous resolution programs driven by specialized pro-resolving mediators (SPMs) acting at pro-resolving receptors (7). As an example, lipoxins synthesized from arachidonic acid during the resolution of self-limited inflammation function as stop signals through the receptor ALX/FPR2 for neutrophil recruitment, facilitate neutrophil apoptosis and efferocytosis (4), pivotal events in efficient resolution. Given the dual nature of inflammation and the importance of the inflammatory response in survival, it is paramount to develop new treatments to overcome the limitations of currently available anti-inflammatory therapies, which are often accompanied with unwanted side effects and do not lead to repair of the affected tissues. Indeed, over the past few years a new branch of pharmacology termed “resolution pharmacology” (8, 9) has emerged, with the goal of developing strategies that promote resolution instead of blocking or inhibiting a mediator or pathway.

This Research Topic brings together thirty-two articles, both reviews and original research papers, which highlight recent advances in resolution biology, wound repair, metabolic disorders, and acquired immunity using a large variety of experimental models. Twenty-four articles report original research on molecular mechanisms that underpin processes that limit inflammation and autoimmune disorders and/or promote tissue repair and return to homeostasis. These studies also provide profiles of the resolution process at the molecular and cellular levels, and discuss new avenues for potential therapeutic interventions based on enhancing pro-resolving mechanisms. Eight reviews summarize current knowledge on the complex molecular and cellular interactions between various immune cells, lipid and protein mediators, and intracellular signaling circuits, which may orchestrate the resolution of a broad spectrum of diseases, including COVID-19, inflammatory and autoimmune diseases, cardiac remodeling, burns, and cancer.

Pro-resolving lipid mediators, including lipoxins, resolvins, maresins and protectins, play central roles in orchestrating efficient resolution (7). Extending previous research, ten articles in this Research Topic discuss recent advances on lipid SPMs, their molecular characteristics, biosynthesis and functions in various pathologies. Libreros et al. describe the synthesis of a new member of the resolvin (Rv) E family, RvE4, its stereochemistry, and capacity to facilitate of the uptake of apoptotic cells (efferocytosis), an essential process to remove damaged cells and inflammatory cells from the inflamed site. Walker et al. report that Protectin Conjugates in Tissue Regeneration 1 (PCTR1) and protectin D1 (PD1) are produced during the resolution of RSV infection and, in turn, reduce viral load while limiting lung type 2 immunity in response to viral infection. Miyata et al. show that during type 2 immunity, activation of 12/15-lipoxygenase is essential for the production of SPMs during IL-33-induced lung inflammation. The SPM products 14(S)-HDoHE and 10(S), 17(S)-diHDoHE markedly reduced ILC2 proliferation and cytokine production to limit IL-33-driven eosinophilic inflammation. Oner et al. report that the SPM RvE1 is a potent inhibitor of Th17 cell differentiation, as well as IL-17 production by acting directly on naïve T cells or indirectly through dendritic cells. Beegun et al. identify dysregulated generation of another potent SPM, maresin 1 during chronic rhino-sinusitis. Maresin 1 reduces expression of adhesion molecules on phagocytes to promote inflammation resolution. Borges et al. demonstrate that adenosine diphosphate, a metabolic product released mainly by platelets at injury sites, plays a role in diabetic wound repair signaling through its receptor P2Y12 to limit inflammatory outcomes. Perez-Hernandez et al. present a critical review on the dual roles of SPM in tilting the effector to regulatory T cell pendulum towards beneficial outcomes and the diagnostic and prognostic value of these mediators in various experimental models and human diseases. Lavy et al. elaborate on this theme by providing an overview on the role of SPMs in the regulation of cancer immunity, with particular emphasis on the role of tumor-associated macrophages. They also discuss the ability of SPMs to re-educate macrophages for combating tumors. Schmid and Brune discuss prostanoids and their emerging functions in resolving inflammation and how they can be used for therapeutic purposes. Paland et al. summarize recent clinical advances in understanding the pathogenesis of COVID-19 and the development of potentially lethal cytokine storm and speculate how phytocannabinoids could be harnessed to treat severe cases and reduce mortality.

In addition to lipid SPMs, protein and peptide mediators have long been recognized as important regulators of resolution. Expanding our knowledge on these mediators, eight publications are dedicated to novel resolution-associated proteins or peptides and provide insight into their function. Martin-Rodriguez et al. describe novel potential therapeutic applications for mediators released by macrophages following efferocytosis in T cell-driven colitis. This secretome, named SuperMApo, notably contains TGF-β, IGF-I, and VEGF that act on intestinal epithelial cells to enhance wound repair and limit tissue damage. SuperMApo single treatment allows the resolution of ongoing experimental colitis. Takahashi et al. report that another wound healing effector, the anti-bacterial peptide beta-defensin 3, can promote angiogenesis, fibroblast migration, and proliferation through FGFR1. Lyngstadaas et al. identified novel functions for the well-known pro-resolving protein annexin A1 acting on conjunctival goblet cells. They report annexin A1 facilitation of calcium influx and mucin secretion from these cells *in vitro*, suggesting a protective role for this protein in inflammatory conjunctival diseases. Pollenus et al. unveil a dichotomic role for the chemokine receptor CCR2 critical for monocyte migration to inflamed and resolving sites in malaria-associated ARDS. They report that while CCR2 is essential for migration of inflammatory and non-classical monocytes during the onset and resolution of inflammation, respectively, CCR2-deficiency has no impact on ARDS development or resolution. Efimova et al. describe obesity as an inflammatory disorder regulated by myeloid cells. They argue for a previously unappreciated role for the glucagon-induced peptide receptor (GIPR) in myeloid cells to maintain a beneficial type II immune response in white adipose tissue. The ECM component syndecan-1, which was previously described as critical for the resolution of inflammation, exerts opposing actions in type II immunity-associated airway remodeling. Zhang et al. show that syndecan-1 enhances the TGF-β/SMAD3 pathway to promote ECM production and myofibroblast generation. Jin et al. review the complex role of chemokines, such as CCL2/MCP-1, and their products in the resolution of inflammation. While most inflammatory chemokines were previously solely considered to be potent pro-inflammatory and autoimmunity-driving effectors, recent data on the genes they induce requires revisiting this notion. For instance, MCP-1-induced peptide 1 (MCPIP1) is highlighted as a key pro-resolving agent that limits NF-kB activation and regulates the stability of specific mRNAs to promote resolution and the clearance of virus-infected apoptotic cells.

The phenotypic and functional heterogeneity of myeloid cells and macrophage subsets in particular, in resolving inflammation and wound repair have received considerable attention during the past years. Tewari et al. describe a novel role for lymph node monocytes in limiting poly I:C-induced, dendritic cell-mediated, T cell cytotoxicity through the induction of IL-10 production and the generation of CD4^+^ suppressive T cells. Consistently, blocking IL-10 in a melanoma-targeted immune response improved the therapeutic potential of poly I:C. Li et al. document another macrophage-regulatory T-cell circuit. This cell-cell interaction limited type 2 immunity by supporting the proliferation of Treg through myeloid production of resistin-like molecule α. Luan et al. show that the pain reliever butorphanol through the opioid κ receptor reduces LPS-induced acute lung injury by shifting the M1-like to M2-like macrophage balance and limiting the function of inflammatory effectors. Krampert et al. report that high sodium environments may hamper the anti-bacterial functions of neutrophils without affecting neutrophil viability. This response is likely mediated through NADPH oxidase-generated ROS and underscores the health hazard associated with high sodium intake. Kemble and Croft discuss the intricate local cell-cell interactions in the inflamed joints. Their overview focuses on synovial resident macrophages and fibroblasts, their mutual regulatory properties, and how their breakdown can lead to persisting pathology. They also discuss how these mechanisms can be harnessed to develop potential therapies. Khan et al. review another regulatory axis between the central nervous system and the spleen. They suggest that this axis is critical in regulating immune responses to thermal injury, and argue that a better understanding of its mechanistic aspects could provide opportunities for new therapies. Kologrivova et al. discuss the role of immune cells in tissue repair with a particular focus on cardiac remodeling and repair following myocardial infarction, with myeloid cells and lymphocytes considered for their dichotomic roles in injury generation and healing.

Six reports provide novel insights into intracellular mechanisms that control key events in inflammation and resolution. Burkard et al. show that desmoglein 2 (Dsg2) regulates the expression of the tight junction protein claudin 2 in intestinal inflammation, and thereby blocks changes in intestinal epithelial barrier induced by inflammatory conditions and PI3 kinase. Dolling et al. document that hypoxia promotes neutrophil survival and reduces NET formation potential following myocardial infarction in humans. The hypoxia-responsive transcription factor HIF-1α seems to be critical in mediating these responses. Cao et al. describe a new axis that governs the inflammatory response in endotoxemia. They identify a critical role for the transcription factor FRA-1 for optimal expression of the anti-inflammatory protein lipocalin 2 during LPS tolerance. Conversely, mice deficient in FRA-1 show deficiencies in resolution. Tsai et al. present evidence supporting a role for aldehyde dehydrogenase 2 (ALDH2) in limiting heat shock-induced endothelial damage and lung injury. The pharmacological stimulator of ALDH2, alda-1, increased enzymatic activity leading to a reduction of ROS production and alleviation of heat exposure-induced damage. Wang et al. reveal that another heat shock protein, αB-crystalline, alleviates LPS-induced uveitis by reducing production of inflammatory cytokines, activation of microglia, autophagy, inflammatory features, and loss of function in the retina. Yan et al. show that the anti-inflammatory transcription factor PPAR-γ limits damage in IgG-immune complex-induced lung inflammation, likely through the inhibition of EGR-1-induced cytokine production.

To illustrate the translational potential of resolution pharmacology, two articles present data from recent clinical studies. In a pilot study, van Heerden et al. report that treatment of patients with mild-to-moderate sepsis with preparations from early apoptotic cells (termed Allocetra™-OTS) led to early resolution of the cytokine storm, and survival of all subjects, as compared with a mortality rate of 27% of historically matched controls. No adverse effects have been observed. Menarim et al. provide detailed transcriptomic and histochemical analysis of bone marrow mononuclear cells recovered during the resolution of osteoarthritis. This report highlights PPAR-γ as a key effector, and hence a promising therapeutic target in resolving synovitis.

In conclusion, this Research Topic presents a selection of articles that underscore the resolution of inflammation as a tightly regulated process involving various lipid and protein effectors predominantly acting through specific surface receptors on responding cells. These effectors, in turn, activate transcriptional cascades that affect gene signatures leading to robust responses. Immune cells, particularly of the myeloid lineage, are key cellular contributors to the resolution of inflammation by regulating their local migration, cytokine production, debris clearance, NETosis, and cell death. Gaining a comprehensive understanding of these processes and identifying novel mediators and pathways that facilitate resolution will likely identify potential targets for development of novel therapeutic approaches for autoimmune, inflammatory, tissue repair, and fibrotic diseases.

## Author Contributions

This editorial was co-authored by all the indicated authors. All authors concur with its content and approve the submission.

## Conflict of Interest

SP is the CEO of MED‘INN’Pharma.

The remaining authors declare that the research was conducted in the absence of any commercial or financial relationships that could be construed as a potential conflict of interest.

## Publisher’s Note

All claims expressed in this article are solely those of the authors and do not necessarily represent those of their affiliated organizations, or those of the publisher, the editors and the reviewers. Any product that may be evaluated in this article, or claim that may be made by its manufacturer, is not guaranteed or endorsed by the publisher.
